# Reproducibility of mild to moderate regurgitation of all heart valves by magnetic resonance imaging

**DOI:** 10.1186/1532-429X-13-S1-P340

**Published:** 2011-02-02

**Authors:** Robert Gradinger, Christina Ring, Thomas Walcher, Wolfgang Rottbauer, Peter Bernhardt

**Affiliations:** 1 Universitiy of Ulm, Ulm, Germany

## Introduction

Cardiac magnetic resonance imaging (CMR) is regarded as the gold standard for evaluation of left and right ventricular volumes and ejection fraction. Assessment of valve regurgitation can be performed by flow measurement in the great arteries for the aortic and pulmonary valve. Subtraction of ventricle stroke volume and flow measurement in the affiliated great artery results in regurgitation volume of the corresponding atrioventricular valve.

Aim of this study was to evaluate the reproducibility of valve regurgitations considering intra- and interobserver variability in patients with mild to moderate valve regurgitation.

## Methods

For sample size estimation two blinded observers analyzed twice the CMR data of ten patients. To yield a confidence interval bandwidth of 0.15 twenty-six patients had to be evaluated by two blinded observers. All images were analyzed by two experienced readers blinded to patients’ data in random order. All patients were analyzed twice by every reader.

## Results

We found good correlations for aortic and pulmonary regurgitation fraction and left ventricular ejection fraction (ICC > 0.9). Correlations for mitral and tricuspid valve regurgitation and right ventricular ejection fraction were lower. Right ventricular ejection fraction: inter-rater reliability: ICC 0.843; intra-rater reliability: rater 1 ICC 0.884; rater 2 ICC 0.840. Mitral regurgitation: inter-rater reliability: ICC 0.875; intra-rater reliability: rater 1 ICC 0.824; rater 2 ICC 0.849. Tricuspid regurgitation: inter-rater reliability: ICC 0.616; intra-rater reliability: rater 1 ICC 0.585; rater 2 ICC 0.787.

## Concusion

Cardiac magnetic resonance imaging offers good to very good inter- and intrastudy agreement for the analysis of mild heart valve regurgitation. However, the exact evaluation of atrioventricular valve regurgitation, especially of the tricuspid valve yield lower inter- and intraobserver agreement. The reason might be low the inter- and intraobserver variability in the calculation of the right ventricular stroke volume, caused by the more complex function and anatomy of the right heart chambers.

